# Supplementary material for: Surface Adsorption Properties and Layer Structures of Homogeneous Polyoxyethylene-Type Nonionic Surfactants in Quaternary-Ammonium-Salt-Type Amphiphilic Gemini Ionic Liquids with Oxygen- or Nitrogen-Containing Spacers
Source: Molecules. 2020 Oct 22;25(21):4881. doi: 10.3390/molecules25214881 (PMC7660069; doi:10.3390/molecules25214881)
Supplement: Supplementary file 1 [file molecules-25-04881-s001.pdf]

Supplementary Materials

Surface Adsorption Properties and Layer Structures of Homogeneous Polyoxyethylene-Type Nonionic Surfactants in Quaternary-Ammonium-Salt-Type Amphiphilic Gemini Ionic Liquids with Oxygen- or Nitrogen-Containing Spacers

Risa Kawai, Maiko Niki, Shiho Yada, Tomokazu Yoshimura*

Department of Chemistry, Faculty of Science, Nara Women's University,
Kitauoyanishi-machi, Nara 630-8506, Japan

*Corresponding author: yoshimura@cc.nara-wu.ac.jp

Details of Measurements

Wide-angle X-ray scattering (WAXS)

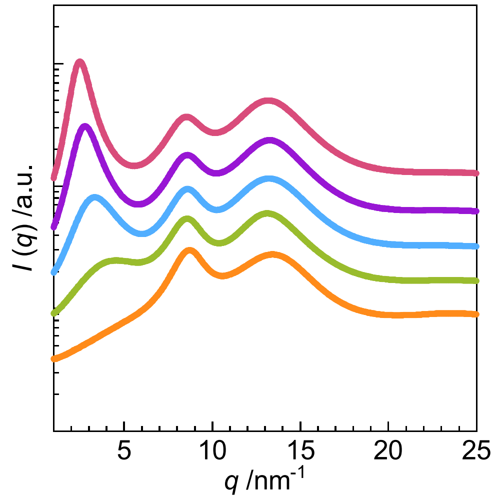

Figure S1. WAXS profiles for amphiphilic monomeric ionic liquids C_n NTf_2 at 25 °C: n = 4 (orange ●), n = 6 (50 °C, yellow green ●), n = 8 (blue ●), n = 10 (purple ●), and n = 12 (50 °C, red ●).

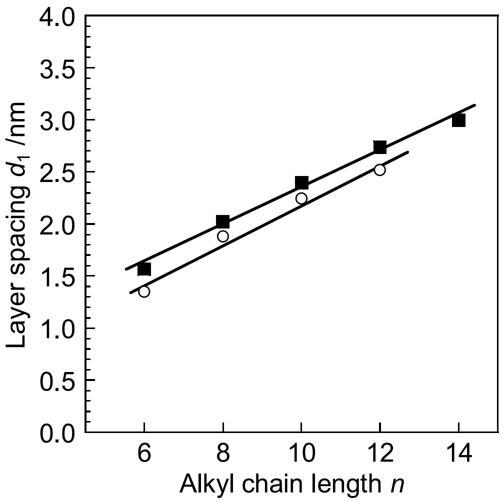

Figure S2. Relationship between layer spacing d_1 and alkyl chain length n for amphiphilic gemini and monomeric ionic liquids at 25 °C: $2C_{12}$(2-O-2) NTf_2 (black ■) and C_n NTf_2 (black ○, 50 °C for n = 6, 10).

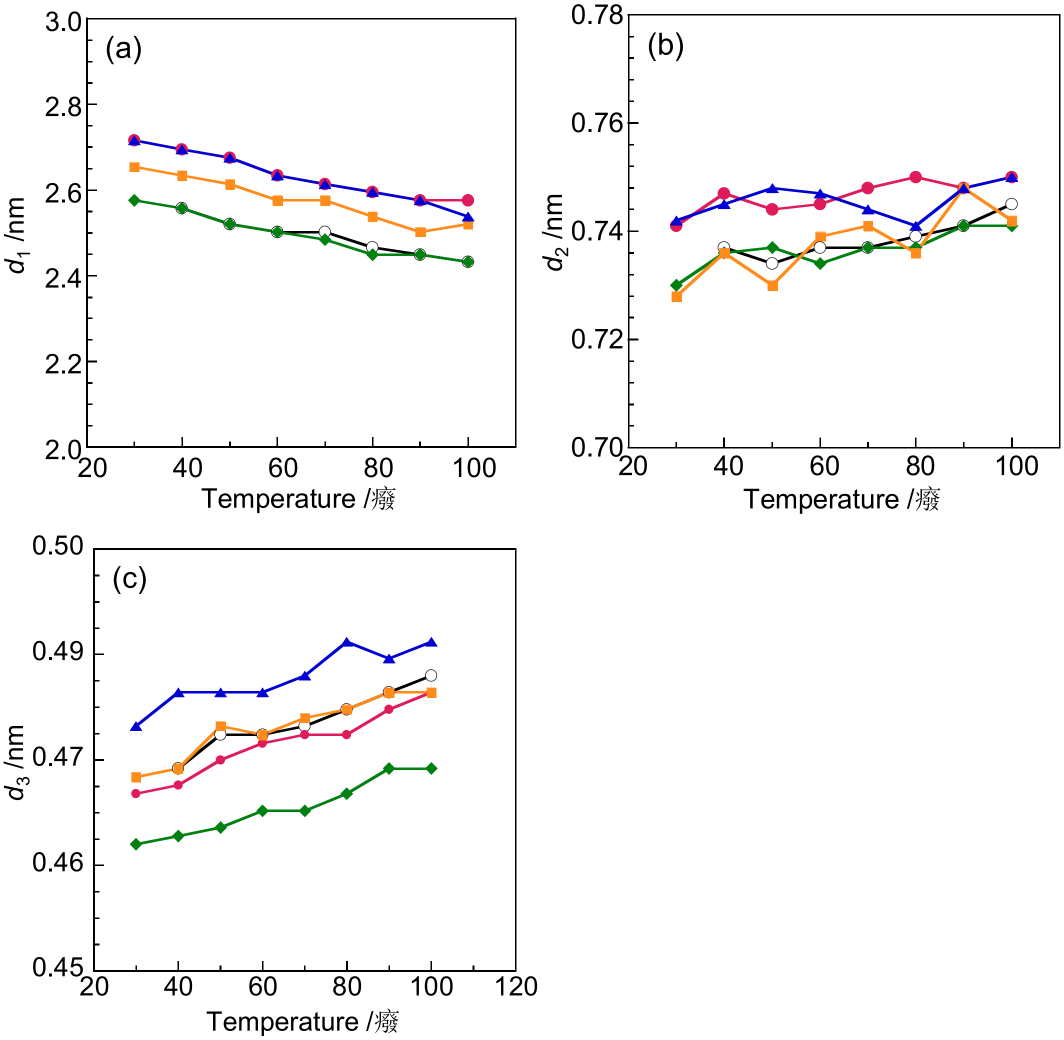

Figure S3. Variation in (a) d_1, (b) d_2, and (c) d_3 with temperature for amphiphilic ionic liquids: C_{12} NTf_2 (black ○), $2C_{12}$(2-O-2) NTf_2 (red ●), $2C_{12}$(2-O-2-O-2) NTf_2 (orange ■), $2C_{12}$(2-N-2) NTf_2 (blue ▲), $2C_{12}$-3 NTf_2 (green ◆).

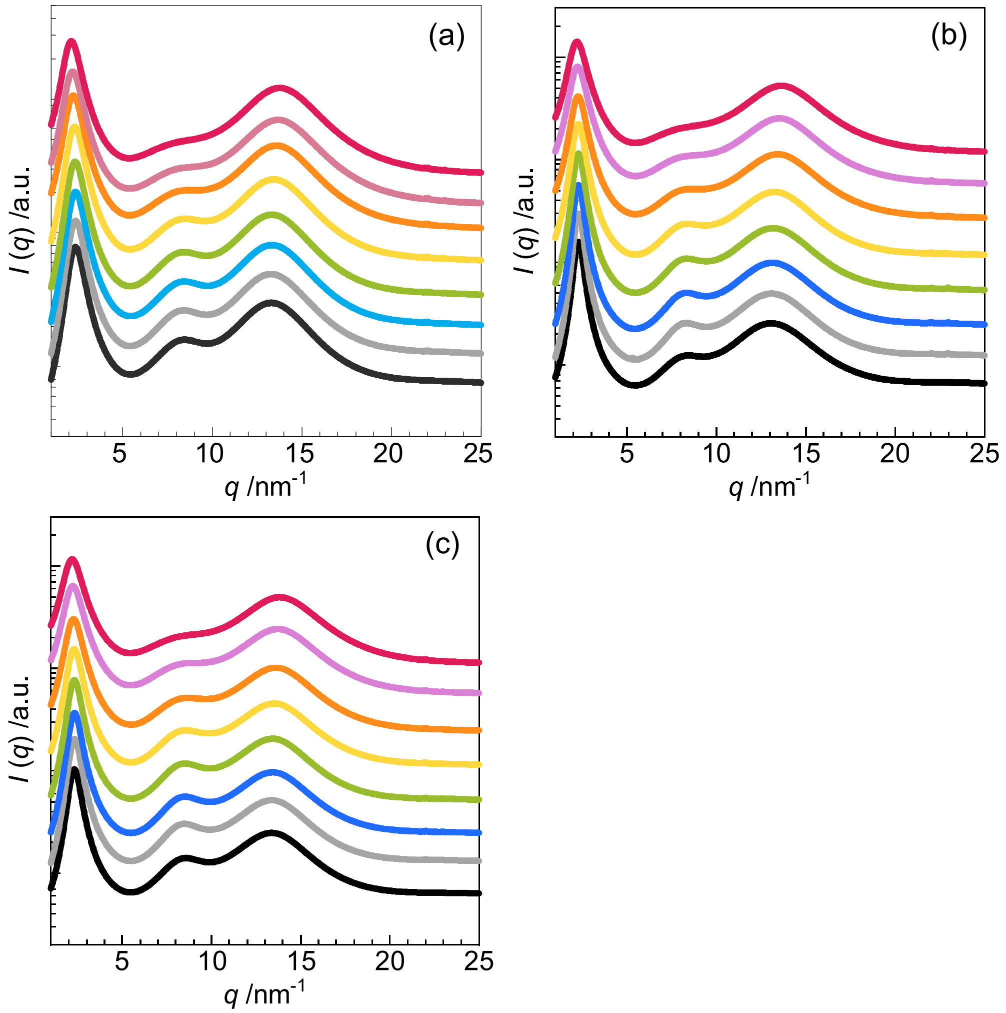

Figure S4. WAXS profiles for nonionic surfactant $C_{12}EO_6$ in amphiphilic gemini ionic liquids at 25 °C: (a) $2C_{12}$(2-O-2-O-2) NTf_2, (b) $2C_{12}$(2-N-2) NTf_2 and (c) $2C_{12}$(2/2-N-2) NTf_2 (30 °C): 0 (black ●), 10 (gray ●), 50 (blue ●), 100 (yellow green ●), 250 (yellow ●), 500 (orange ●), 750 (pink ●), and 1000 mmol dm^{-3} (red ●).

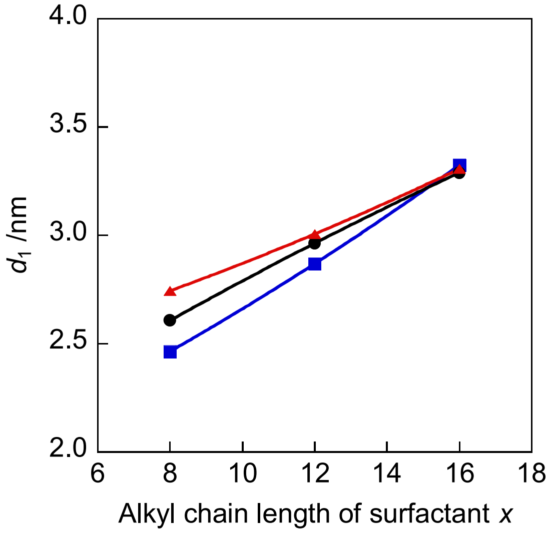

Figure S5. Relationship between d_1 and alkyl chain length of nonionic surfactants C_xEO_6 at 1000 mmol dm^{-3} in amphiphilic gemini ionic liquids: $2C_{10}$(2-O-2) NTf_2 (blue ■), $2C_{12}$(2-O-2) NTf_2 (black ●), and $2C_{14}$(2-O-2) NTf_2 (red ▲).

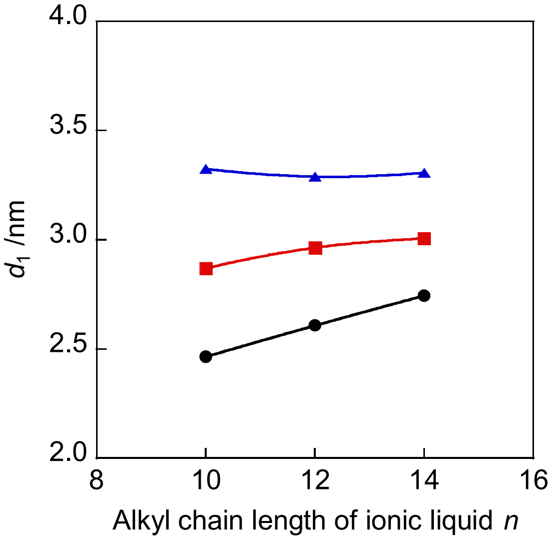

Figure S6. Relationship between d_1 and alkyl chain length n of amphiphilic ionic liquid $2C_n$(2-O-2) NTf_2 containing nonionic surfactants C_xEO_6 at 1000 mmol dm^{-3}: C_8EO_6 (black ●), $C_{12}EO_6$ (red ■), and $C_{16}EO_6$ (blue ▲).

Cryogenic transmission electron microscopy (cryo-TEM)

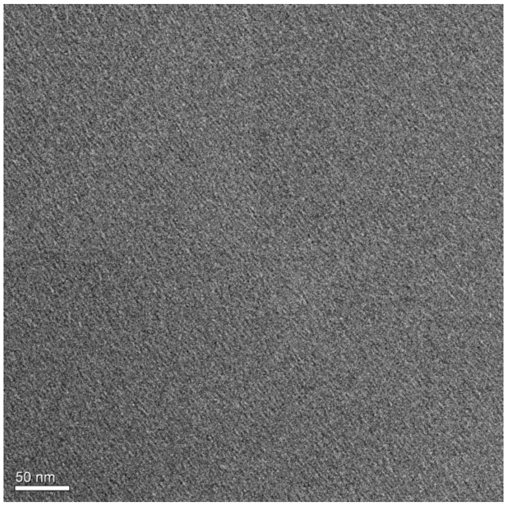

Figure S7. Cryo-TEM image of 750 mmol dm^{-3} $C_{12}EO_6$ in $2C_{12}(2\text{-}O\text{-}2)$ NTf_2.
